# Actin Dynamics at the T Cell Synapse as Revealed by Immune-Related Actinopathies

**DOI:** 10.3389/fcell.2021.665519

**Published:** 2021-06-24

**Authors:** Loïc Dupré, Kaan Boztug, Laurène Pfajfer

**Affiliations:** ^1^Ludwig Boltzmann Institute for Rare and Undiagnosed Diseases (LBI-RUD), Vienna, Austria; ^2^Department of Dermatology, Medical University of Vienna, Vienna, Austria; ^3^Toulouse Institute for Infectious and Inflammatory Diseases (INFINITy), INSERM, CNRS, Toulouse III Paul Sabatier University, Toulouse, France; ^4^St. Anna Children’s Cancer Research Institute (CCRI), Vienna, Austria; ^5^CeMM Research Center for Molecular Medicine of the Austrian Academy of Sciences, Vienna, Austria; ^6^Department of Pediatrics and Adolescent Medicine, Medical University of Vienna, Vienna, Austria; ^7^St. Anna Children’s Hospital, Department of Pediatrics and Adolescent Medicine, Medical University of Vienna, Vienna, Austria

**Keywords:** T lymphocytes, immune-related actinopathies, actin cytoskeleton remodeling, immunological synapse, actin binding proteins, primary immunodeficiencies

## Abstract

The actin cytoskeleton is composed of dynamic filament networks that build adaptable local architectures to sustain nearly all cellular activities in response to a myriad of stimuli. Although the function of numerous players that tune actin remodeling is known, the coordinated molecular orchestration of the actin cytoskeleton to guide cellular decisions is still ill defined. T lymphocytes provide a prototypical example of how a complex program of actin cytoskeleton remodeling sustains the spatio-temporal control of key cellular activities, namely antigen scanning and sensing, as well as polarized delivery of effector molecules, via the immunological synapse. We here review the unique knowledge on actin dynamics at the T lymphocyte synapse gained through the study of primary immunodeficiences caused by mutations in genes encoding actin regulatory proteins. Beyond the specific roles of individual actin remodelers, we further develop the view that these operate in a coordinated manner and are an integral part of multiple signaling pathways in T lymphocytes.

## Preamble

The actin cytoskeleton provides a structural basis to sustain, not only cell shape remodeling, but also nearly every dynamic cellular process. The actin cytoskeleton is endowed with highly dynamic and adaptable properties. Indeed, multiple actin networks that are uniquely assembled coexist within cells, occupying specific locations and exerting specialized cellular processes (Blanchoin et al., 2014; Lappalainen, 2016). These distinct networks are governed by different dosage of the multiple molecular activities that tune actin dynamics (Pollard, 2016). Interestingly, the diversity of actin-driven processes reflects the multiplicity of dynamic activities shared among all cells, as well as the numerous specialized activities at work in individual cell subsets. This notion applies particularly to the hematopoietic system, which is composed of cells with highly diversified and specialized activities that rely on actin dynamics.

In particular, T cells assemble the immunological synapse (IS), an adhesive structure that is the site of complex actin network organization and dynamics. As such the T cell synapse provides a prototypical example of how the actin cytoskeleton sustains the coordination of key cellular activities, including adhesion, receptor patterning and control of secretory events, to name a few. The requirement of actin cytoskeleton integrity for productive T cell/APC encounter and for the assembly of key signaling networks has been established along with the characterization of the IS (Billadeau et al., 2007). Recent studies have started to apply resolutive approaches to uncover some of the complexity of actin remodeling at this highly specialized structure. The current notion is that of multiple supportive roles for the actin cytoskeleton at the T cell synapse. From a spatial perspective, sub-networks have been identified, with specific interactions with receptor clusters that display specific patterning. From a temporal perspective, actin remodeling supports a sequence of events that are particularly crucial to tune T cell activation and function.

The many facets of actin cytoskeleton remodeling are supported by a multiplicity of molecular actors that integrate complex stimuli into adapted actin responses. Research in this field has been shaped by the discovery of rare deficiencies, which highlight the role of specific molecules in the function of immune cells. In particular actin-related primary immunodeficiencies (PID) might be considered as models to investigate the tuning of T cell activation and function by the actin cytoskeleton. This review covers our current understanding of the molecular control of actin cytoskeleton remodeling at the T cell IS via the prism of actin-related PID. By now, approximately 20 PID entities due to actin defects (so called actinopathies) have been identified (Burns et al., 2017; Janssen and Geha, 2019; Tangye et al., 2019). These rare pathologies provide unique models to reveal the physiological role of actin regulators in human T cells in the context of natural environmental challenges.

Here we provide a review on actin dynamics and sub-networks supporting the T cell IS. We then highlight how the elucidation of actin-related PID has led to fundamental discoveries about the molecular regulation of the actin cytoskeleton at the T cell IS. Then, we try to integrate knowledge from individual studies around key aspects of actin dynamics at T cell IS. In particular, we discuss recent research about how actin regulators coordinate their activities to assemble specific actin networks. We also discuss how distinct actin networks may combine their function to control the sequential steps of the T cell IS.

## Control of the T Cell Immunological Synapse by the Actin Cytoskeleton

### Multilayered Activities of the Actin Cytoskeleton at the Immunological Synapse

**FIGURE 1:**
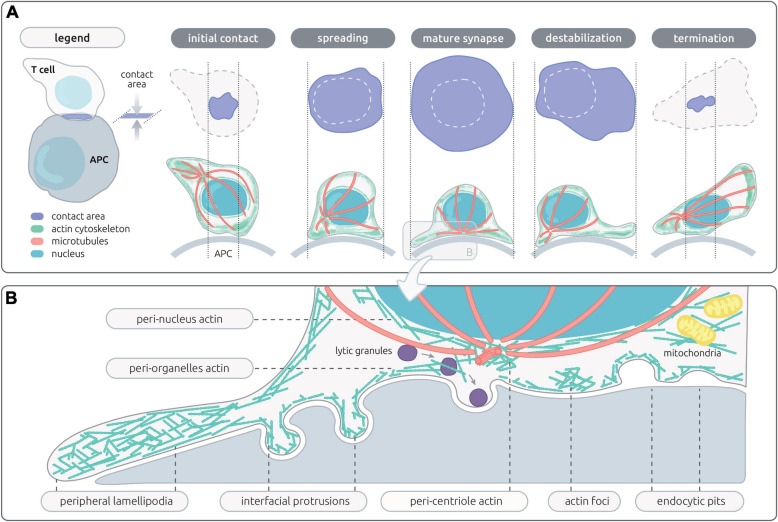
Actin networks sustaining T cell synapse shape and microarchitecture. **(A)** Schematic representation of the en-face and side views of the contact area between a T cell and an antigen-presenting cell (APC). The successive steps of the IS life cycle are depicted, from initial T cell/APC contact to termination of the IS. The contact area, actin cytoskeleton, microtubules and nucleus are represented with the indicated colors. **(B)** Schematic representation of the microarchitecture of a mature IS, corresponding to a transversal view of the contact between a CD8^+^ T cell and a target cell. This representation assembles the different actin networks described so far in the context of lymphocyte activation, including those proximal to the plasma membrane and those associated with inner organelles of the cell.

Through its intrinsic remodeling properties, the actin cytoskeleton provides a structural basis for the dynamic and polymorphic architecture of the IS. The fact that actin cytoskeleton integrity is necessary to establish dynamic and prolonged contacts between T cells and antigen-presenting cells (APC) (Valitutti et al., 1995; Delon et al., 1998) was actually recognized prior to the characterization of the molecular organization of the IS (Monks et al., 1998; Grakoui et al., 1999). Following these early observations, a wealth of studies has contributed to the current notion that the actin cytoskeleton exerts multiple tasks during the T cell/APC interaction. These tasks may be split as those operating at the cellular scale (Figure 1A) and those operating at the mesoscale (Figure 1B). At the cellular scale, the actin cytoskeleton promotes a morphological transition from an elongated shape characterizing migrating lymphocytes to a round-up shape following adhesion to the APC and migration arrest (Negulescu et al., 1996). The stop signal is delivered through TCR evoked phosphorylation of myosin IIA, which inactivates the contractile machinery responsible for rapid motility (Jacobelli et al., 2004). The actin cytoskeleton then sustains the formation of lateral membrane protrusions that scan the surface of the APC (Tskvitaria-Fuller et al., 2003). At the mesoscale, it is remarkable that the local architecture of the actin cytoskeleton differs along the radial layers of the IS (recently reviewed in Roy and Burkhardt, 2018; Hammer et al., 2019). The first identified sub-structure has been the dynamic ring-like structure at the periphery of the synapse, which is enriched in branched F-actin and corresponds to a radial lamellipodium sought to stabilize the IS (Bunnell et al., 2001). Quite distinctly, the center of the T/APC contact is characterized by a relative depletion of F-actin. Although it had initially been considered a region deprived of F-actin (Stinchcombe et al., 2006), improved resolution has revealed that the IS center is sustained by an actin network with loose reticulation. The partial depletion of F-actin at the IS center is reached within one minute of IS assembly so that it has been proposed to initiate key subsequent events (Ritter et al., 2015). These include the polarization of the MTOC (Stinchcombe et al., 2006) and the delivery of secretory vesicles such as lytic granules (Brown et al., 2011; Rak et al., 2011). Beyond these distinct F-actin subdomains, actin cytoskeleton integrity is essential to assembly of TCR microclusters and signaling platforms (Campi et al., 2005). Furthermore actin dynamics is characterized by an inward flow promoting the centripetal transport of receptor microclusters (Varma et al., 2006; Kaizuka et al., 2007). Interestingly, differential coupling of surface receptors promotes molecular segregation into distinct domains, such as TCR (cSMAC) and LFA-1 (pSMAC). The actin cytoskeleton is also playing an active role in TCR endocytosis and recycling (McGavin et al., 2001). It may seem puzzling how the actin cytoskeleton drives so many distinct activities in the confined space of the IS. Such functional multiplicity and diversification relies on the ability of the actin cytoskeleton to build local networks of specific filament length, density and reticulation. As we will see in the following chapter dedicated to natural deficiencies in actin regulatory proteins, these local networks are governed by specific molecular activities. The plethora of activities sustained by the actin cytoskeleton is also intimately related to the dynamic nature of such networks and the recycling nature of actin remodeling. Another key property of the actin cytoskeleton is its ubiquitous ability to interact with membranes, receptor complexes and organelles. The actin cytoskeleton is indeed endowed with the capacity to adapt its structure to the encountered physical constrains. Those considerations regarding the actin cytoskeleton remodeling properties certainly are not restricted to the IS and apply in numerous other cellular processes. However, the IS provides a prototypical example of how multiple layers of physical and biochemical events can be coordinated in a restricted space. We will review in the next sections the current knowledge on the actin-controlled IS events at the cellular and meso-scales.

### Actin Control of the Immunological Synapse at the Cellular Scale: Shape, Symmetry, and Polarity

#### Shape Remodeling Along the Immunological Synapse Life Cycle

The IS is established very rapidly upon recognition of cognate APC. It is driven by a spreading behavior over the surface of the APC (Figure 1A), which is sustained by actin polymerization generating forces to appose the membrane of the T cell to that of the APC. Then T cells further emit a radial extension beyond the cell body, in the form of an undulating lamellipodium. Actin remodeling is among the most early molecular activity downstream of TCR stimulation, as shown by a recent phosphoproteomic analysis (Locard-Paulet et al., 2020). This is in line with early findings that the initial actin cytoskeleton response precedes calcium flux (Delon et al., 1998). Actin-driven membrane dynamics seems to operate through successive steps. The application of lattice light-sheet microscopy has allowed gaining high spatial and temporal resolution to study the transition from migration to IS assembly and maturation (Ritter et al., 2015). Upon contact of migrating murine CD8^+^ T cells with APC, a collapse of the uropod is first observed, while actin-based ruffles and projections emerging from the leading edge move in a reward actin flow toward the back of the cell. Rapidly then the central part of the IS harbors a relatively poor content in F-actin. The TCR molecules, initially at the uropod, accumulate within 2 min of target cell interaction initially via lateral translocation and in a second wave via the intracellular vesicle pool. With a further delay of 6 min in average, the MTOC repolarizes from the uropod to the center of the IS. When CTLs detach from the target cell, they reverse their polarity by initiating an actin-rich lamellipodia at the distal pole, with the centrosome, granules, and TCR relocalizing to the newly formed uropod (Ritter et al., 2015).

#### The Different Shades of Immunological Synapses

The IS is usually depicted as a ≪ model synapse ≫, with perfectly radial organization, important spreading and clear-cut segregation of sub-domains. However, there is a wide dispersion in IS shapes and symmetries across T cell populations and depending on stimulation systems. A majority of the work assessing T cell IS organization is based on planar APC substitutes, by either coating stimulatory molecules on glass or plastic or by embedding stimulatory molecules in lipid bilayers. These systems obviously present the advantage of being able to control stimulatory molecule identity and density as well as to image the IS with high resolution. In the context of T cell/APC interactions, differences in IS assembly are expected due to ligand composition and densities, ligand dynamics and anchorage, surface structure and biophysical properties. There are multiple shades between the migratory behavior of T cells and the full arrest upon cognate APC encounter, reflecting the fact that T cells scanning for antigens are sensing concurrent stop and go signals such as chemokines (Dustin, 2004; Viola et al., 2006). This has led to the concept that T cells may assemble either stable synapse or motile IS, named “kinapses” (Dustin, 2008). This would match the notion that T cell/DC encounters might be highly heterogenous in length, from few minutes to hours (Gunzer et al., 2000; Mempel et al., 2004; Miller et al., 2004).

The existence of intermediate states infers that migration and IS conformation might not be drastically opposing behaviors. As proposed by Dustin, stabilization of the IS might not correspond to a shut-down of the motility machinery but rather a symmetrization of force-generating structures to balance forces and hold the cell in contact with the APC (Dustin, 2008). IS turnover and T cell detachment would then result from a mechanism of symmetry breaking. This view is comforted by the fact that migrating CTL and those forming conjugates have remarkably similar actin dynamics, including projections forming at the leading edge and actin flowing rearward toward the uropod (Ritter et al., 2015). The attractive notion about this view is that graded tuning of stabilization might provide a mechanism to adjust T cell scanning and resulting activation.

*In vivo* imaging of tagged TCR in naïve T cells provided evidence that TCR clustering and internalization is not strictly dependent on the establishment of stable contacts with APC (Friedman et al., 2010). Using *in situ* cytometry, Moreau et al. have found that TCR-pMHC affinity controls T cell motility during antigen recognition *in vivo*. In particular in the context of intermediate affinity ligands, kinapses are the predominant form of APC contacts and are able to sustain strong TCR signals (Moreau et al., 2012). Actin remodeling, in particular via the Arp2/3 complex, appears necessary to drive the stop behavior leading to stable IS, but dispensable for the partial deceleration characteristic of the kinapse (Moreau et al., 2015). This suggests that distinct actin remodeling patterns tune the ≪ appetite ≫ of T cells to the antigenic stimulus by favoring motile scanning of APC with low stimulatory capacity and full arrest on highly stimulatory APC.

### Actin Control of the Immunological Synapse at the Mesoscale: Local Meshworks, Receptor Clusters, Organelle Traffic

#### Local Actin Networks and Meshworks

One key to the understanding of the plethora of functions sustained by the actin cytoskeleton at the IS is its organization in discrete dynamic networks (recently reviewed by Hammer et al., 2019). These networks correspond to substructures that are not unique *per se* to T cells, but that are arranged in a unique fashion at the T cell IS (Figure 1B). The most prominent networks comprise a lamellipodium that tend to arrange radially, a lamellar connecting the lamellipodium to the cell body and multiple actin foci distributed at the IS interface. These networks are segregated in space but are also structurally distinct as they are shaped by different actin filament length and branching degree. Synapse assembly and maturation is supported by the sequential organization of these distinct networks, as recently revealed by live 3D STED of Jurkat T cells (Fritzsche et al., 2017). The first step of spreading is supported by the ruffles decorating resting cells, which is followed by assembly of a flat undulating lamellipodium supported of a dense actin filament meshwork. Within a few minutes, the lamellipodium starts contracting. Below the cell body, both a cortical network as well as a cytoplasmic network can be distinguished and are separated from the lamellipodium by a ring enriched in myosin-II. Remarkably, both an inward-growing ramified actin network and an outward-growing lamellipodial network co-exist Jurkat T cells forming 2D synapses. The relative importance of these networks as well as their dynamic properties seem, however, to differ between Jurkat T cells and primary T cells (Colin-York et al., 2020), and possibly among different T cell subsets.

Beyond those dynamic networks that sustain the main subdomains of the IS, the actin cytoskeleton sustains different types of membrane protrusions. It has been long appreciated that T cells are covered with actin-rich microvilli that represent the first surface T cells use to scan their environment. Recent studies point to the importance of these tiny structures as structural units to preassemble receptors and proximal signaling molecules and as structural basis for the “palpation” of the APC surface and scanning of pMHC complexes by the TCR (Jung et al., 2016; Cai et al., 2017; Ghosh et al., 2020). Although the apposition of the plasma membrane from the T cell and the APC is depicted as flat, there is evidence for indentations in the form of podosome-like structures. This applies to antigen-experienced T cells inspecting the surface of endothelial cells and APC (Carman et al., 2007; Sage et al., 2012) and to CTL interacting with target cells (Tamzalit et al., 2019). How actin dependent microvilli and larger membrane indentations may relate to the underlying dynamics actin cortex is currently unclear.

#### Receptor Patterning and Signaling via Differential Coupling to the Centripetal Actin Flow

The centripetal actin flow has been established as a key driver of cell surface receptor dynamics at the IS. Via differential anchorage to the cortical actin, receptors are transported towards the IS center at different rates (reviewed in Beemiller and Krummel, 2010). This transport mechanism allows the typical molecular patterning of the IS characterized by the positioning of TCR at the cSMAC, adhesion molecules as a ring at the pSMAC and exclusion of large molecules such as CD45 at the dSMAC. The centripetal movement of TCR microclusters is a key rheostat to T cell activation since it provides a balance between sustained TCR signaling and TCR internalization/degradation (Lee et al., 2003; Barda-Saad et al., 2005; Campi et al., 2005; Varma et al., 2006). Interestingly the cSMAC may accommodate both signaling and degradation depending on antigen quality (Cemerski et al., 2008). Whether this is related to distinct rates of actin-driven TCR internalization has not been elucidated. Beyond this large-scale molecular segregation, the anchorage to the actin cytoskeleton allows for force generation, which is an integral mechanism of receptor activation applying to the TCR, integrins and probably many co-receptors (Bashour et al., 2014; Das et al., 2015; Hu and Butte, 2016; Li and Springer, 2017). Remarkably, antigen unbinding kinetics have recently been shown to impact the actin retrograde flow velocity, in such a way that T cells appear to normalize TCR force generation across various antigens (Colin-York et al., 2019). Such feed-back mechanism would allow T cells to widen the range of antigens able to evoke productive TCR engagement. While TCR are held in the cSMAC of mature IS, the active conformation of integrins is confined to the pSMAC (Comrie et al., 2015). The differential motility behavior of TCR and integrins in relation to the underlying actin cytoskeleton probably reflects distinct mechanical coupling. This is supported by the observation that TCR is associated to actin foci (Kumari et al., 2015), while LFA-1 clusters appear to align with the lamella-like region of the IS, which presents with a high acto-myosin activity, taking the shape of linear filaments organized into concentric arcs in Jurkat T cells (Murugesan et al., 2016). This receptor-specific coupling is supported by the identification of distinct actin machineries for TCR and LFA- 1 clusters (driven respectively by Arp2/3 and formins) (Tabdanov et al., 2015). A further layer of regulation arises from the fact that engagement of integrins may constrain the centripetal actin flow, thereby altering signaling microcluster motion and TCR signaling (Nguyen et al., 2008; Jankowska et al., 2018).

Engagement of costimulatory molecules such as CD28 has since the first descriptions of the IS been appreciated to promote actin polarization as well as redistribution of receptors towards the T cell:APC interface (Wülfing and Davis, 1998). The actin remodeling activity of CD28 has recently been shown to operate via the stimulation of WAVE2 and cofilin (Roybal et al., 2016). A further example of how co-receptors impact molecular organization at the IS via their anchorage to the actin cytoskeleton is given by CD2, which assembles as a corolla towards the edge of the IS via its cytoplasmic tail involved in F-actin coupling. At this site, CD2 has been recently proposed to capture other costimulatory molecules, such as CD28, ICOS, CD226 and SLAM-F1 (Demetriou et al., 2020). CD2 is also involved in the localization of the inhibitory receptor PD-1 at the IS, as well as its negative impact on signaling. Interestingly, PD-1 blocks the TCR-induced stop signal (Fife et al., 2009), reinforcing the notion that co-receptors modulate T cell activity at least in part via the regulation of IS assembly and stability. As stated above, such regulation is expected to operate via specific coupling to the actin cytoskeleton, as further exemplified by a recent study showing that PD-1 promotes actin remodeling at the IS via cofilin, coronin and Arp2/3 (Ambler et al., 2020).

The ERM (Ezrin/Radixin/Moesin) protein family stems as particularly relevant to the coupling of T cell surface receptors to the cortical actin. Indeed these proteins crosslink transmembrane proteins and actin filament upon conformational activation that is initiated locally by PIP2 (Fehon et al., 2010; McClatchey, 2014). In T lymphocytes, only ezrin and moesin are expressed (Shaffer et al., 2009), and are involved in migration, adherence, and IS formation. In particular, ezrin and moesin have been shown to drive CD43 clearing from the center of the IS (Allenspach et al., 2001; Delon et al., 2001), to regulate TCR patterning (Roumier et al., 2001), as well as to contribute to signaling microcluster dynamics and patterning (Lasserre et al., 2010). ERM proteins have also been recently described as effective sensor at the initial stage of TCR signaling (Ghosh et al., 2020). More precisely, T cells possess thin actin rich structures called microvilli, enriched in TCR, CD2, coreceptors, LAT and Lck molecules as well as phosphorylated EMR protein (Brown et al., 2003; Ghosh et al., 2020). The TCR molecules are indeed localized at the microvilli thanks to their interaction with the ERM proteins and microvilli then act as hubs for TCR signaling.

In conclusion, the dynamic and adjustable scaffold provided by the cortical actin network at the IS sustains key biophysical properties essential for antigen sensing and signal integration via the TCR, integrins and numerous co-receptors. It sustains molecular transport and force exertion during receptor scanning, as well as molecular segregation for signal organization, amplification and termination.

#### Actin-Microtubule Interplay at the Immunological Synapse

A key event in the regulation of T cell polarity in the context of IS assembly is the translocation of the centrosome from the uropod towards the T cell/APC interface (Geiger et al., 1982). As explained above, this event occurs rapidly after the partial clearance of F-actin from the IS center (Ritter et al., 2015). The actin and microtubule cytoskeletons are coordinated at the IS via a bidirectional interplay. MTOC translocation to the IS depends on phosphorylation of PLC–g1 (Quann et al., 2009), which is stimulated by F-actin polymerization (Babich et al., 2012). Reversely, centrioles play a key role for shaping F-actin remodeling at the IS since their genetic deletion leads to impaired formation of the F-actin peripheral ring and loss of F-actin-dependent force exertion towards the target cells in the context of CTL (Tamzalit et al., 2020). Below the IS, some of the microtubules irradiating from the MTOC distribute parallel to the synapse plane and appear to anchor in the peripheral actin ring. Such anchorage has been proposed to assist MTOC polarization towards the IS center and to be regulated by microtubule linkers such as IQGAP-1 and ezrin (Kuhn and Poenie, 2002; Lasserre et al., 2010). An additional layer of coordination between the actin and tubulin cytoskeletons might be provided by the F-actin cloud surrounding the centrosome. This distinct centrosomal actin network dependent on the Arp2/3 activity and has recently been characterized in the context of B cells in which it controls the polarization of the centrosome to the IS and regulates microtubule growth (Obino et al., 2016; Inoue et al., 2019). This network is also relevant to the T cell IS, where its disassembly is a prerequisite to MTOC polarization (Bello-Gamboa et al., 2020).

#### Actin Dynamics Controlling Endocytic and Exocytic Events at the T Cell Synapse

Endocytic and exocytic events occurring at the IS are key to fuel receptors and signaling molecules, to regulate receptor endocytosis, degradation and recycling, as well as to control the polarized delivery of effector molecules such as lytic molecules, cytokines and chemokines (recently reviewed in Mastrogiovanni et al., 2020). The traffic of vesicular compartments within the cell and at the plasma membrane depends on highly complex and specialized molecular machineries, as well as the coordinated action of the actin and microtubule cytoskeletons. This applies to the assembly of TCR microsclusters, which depend at least in part from intracellular pools of TCR and signaling molecules such as Lck and LAT (Balagopalan et al., 2018). It also applies to the internalization and endosomal trafficking of multiple key receptors. A clear illustration of the role played by specific actin remodeling in the endosome-to-membrane recycling processes is provided by the finding that T cells lacking the Arp2/3 activator WASH fail to maintain surface levels of the TCR, CD28, LFA-1 and GLUT1, as a result of a collapse of the endosomal compartment (Piotrowski et al., 2013).

The role of the cytoskeleton in the control of the polarized delivery of lytic granule content to the IS has been a matter of debate. The prevalent model has been that, upon TCR activation, lytic granules cluster around the centrosome so that centrosome translocation to the IS brings the core of the lytic granules towards the center of the IS (Ritter et al., 2015). The apposition of the centrosome to the plasma membrane may even sustain direct delivery of lytic granules for membrane docking and fusion (Stinchcombe et al., 2006). However, the dependency of lytic granule polarized secretion to centrosome reorientation is not that strict as evidenced by studies showing that the centrosome might be dispensable for the directional secretion of lytic granules (Bertrand et al., 2013; Tamzalit et al., 2020). CTL are particularly dependent on the assembly of a tight junction at the synapse with target cells via the LFA-1 belt at the pSMAC which has been proposed to act as a seal to confine lytic granule content delivery towards the target cell (Somersalo et al., 2004; Le Floc’h et al., 2013). At the cSMAC, F-actin is not deprived as initially sought but assembled into an Arp2/3-dependent branched network, which acts as a filter to regulate lytic granule exocytosis. Degranulation is indeed dependent on local F-actin meshwork clearances controlled via coronin-1A activity (Mace and Orange, 2014). Lytic granule presence and such clearances might not be coordinated, but rather stochastic clearance formation and disappearance appear to provide a mechanism to increase the probability for granule exocytosis (Carisey et al., 2018). Furthermore CTL apply mechanical force against the target cell. This is coordinated in time and space with lytic granule secretion and allows boosting the lytic activity of perforin (Basu et al., 2016). Interestingly, once degranulation of lytic granules has occurred, actin density recovers across the synapse as a mechanism to halt further secretion (Ritter et al., 2017).

A key effector function of T cells is their ability to produce and secrete a vast array of cytokines, whose nature depends on the specification of T cells towards differentiated subsets. Interestingly, cytokines might be secreted at the IS in a polarized manner or released multi-directionally (Huse et al., 2006), implying that distinct secretory machinery are responsible for the delivery of cytokines in targeted versus diffusive modalities. The dependency of cytokine secretion to the actin cytoskeleton machinery has not been investigated as thoroughly as for lytic granules. Cytokines transit from the Golgi apparatus to the plasma membrane through distinct vesicles. Secretion of cytokines by CD4^+^ T cells requires Cdc42-driven actin remodeling but not MTOC polarization (Chemin et al., 2012). In particular defective polarized secretion of IFN-γ was related to a reduced ability of Cdc42-silenced T cells to deplete F-actin at the center of the IS established with super-antigen-pulsed B cells. Whether local actin clearance is regulated as for lytic granules is currently unknown. It is tempting to speculate that cytokine release might be differentially controlled by the actin cytoskeleton, depending on vesicle types and T cell subsets in a way to balance polarized versus diffusive secretion, that may correspond to distinct biological scopes (selective activation of DC and help to B cells via polarized cytokine delivery, versus bystander activation and diffusive delivery of inflammatory mediators).

In addition to the controlled release of effector molecules, TCR stimulation induces the release at the synapse of extracellular microvesicles containing TCR, CD40L, ICAOS and tetraspanins (Blanchard et al., 2002; Choudhuri et al., 2014). Such event may buffer T cell activation and modulate DC stimulation and B cell help. Cytotoxic lymphocytes have recently been shown to also release supramolecular attack particles as autonomous killing entities (Bálint et al., 2020). Whether the synaptic release of these various extracellular microvesicles depends on the actin cytoskeleton remains to be defined.

#### Additional Cytoplasmic Pools of Actin Assist Positioning and Dynamics of Major Organelles

Immunological synapse assembly is accompanied by cytoskeleton-dependent mitochondrial redistribution towards the T cell-APC interface. This localization allows maintaining Ca^2+^ influx across the plasma membrane and downstream activation (Quintana et al., 2007). Furthermore, platinum replica electron microscopy has recently revealed that mitochondria are embedded in a dynamic actin-Myosin-II cytoskeleton network assisting constriction and fission (Yang and Svitkina, 2019). Such events are key to regulate metabolic switches along T cell differentiation and reactivation (Buck et al., 2016). Whether such events occur at and are dependent on the IS is currently unknown.

Nuclear A-type lamins that form the nuclear lamina have been found to be upregulated in activated T cells and to promote F-actin polymerization via the linker of nucleoskeleton and cytoskeleton (LINC). Furthermore lamin-A expression was associated with TCR clustering and increased signaling (González-Granado et al., 2014). A structural link between the nucleus and the IS via a lamin-A-LINK-actin axis might provide a mechanism allowing direct transmission of mechanical forces from the plasma membrane to the nucleus. Whether this might modulate T cell transcriptional activity as reported in other cellular models (Wang et al., 2009) remains to be investigated.

## Molecular Control of Actin Remodeling at the Immunological Synapse via the Prism of Actin-Related Pids

### T Cell Immunological Synapse Defects Caused by Deficiencies in the Actin-Binding Proteins ARPC1B, HEM1, WASP, WIP, and WDR1

**FIGURE 2:**
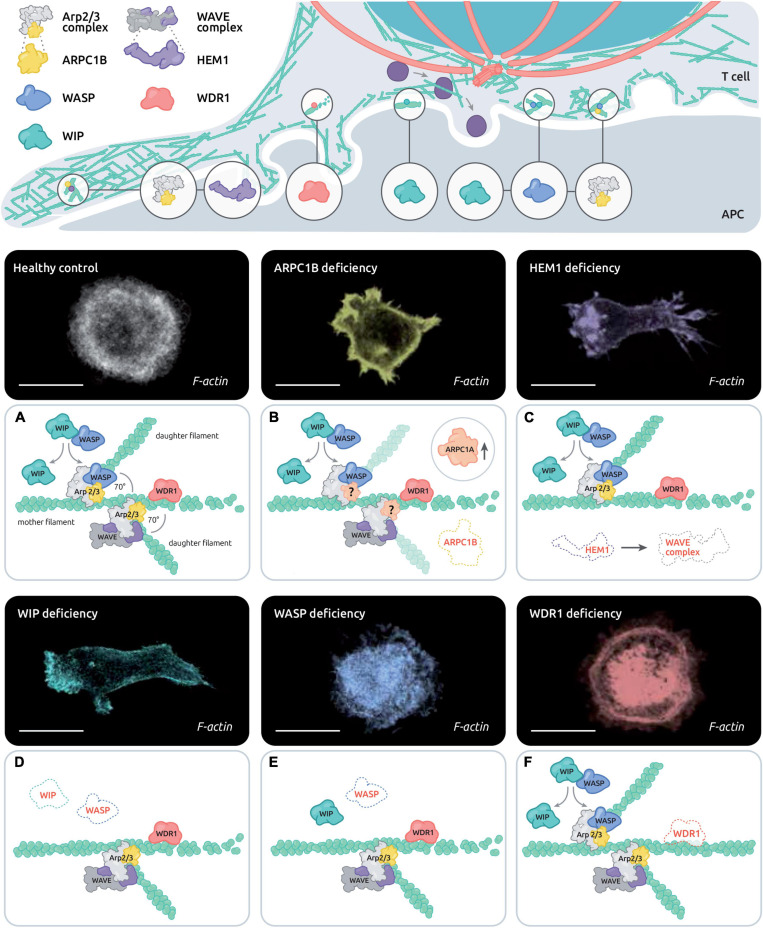
Control of actin remodeling at the T cell synapse by the disease-related actin-binding proteins ARPC1B, HEM1, WASP, WIP and WDR1. The upper scheme depicts the estimated distribution of PID associated actin-binding proteins in the various actin networks of the IS. The lower panels combine representative T cells stained with phalloidin and the corresponding molecular alterations in the context of healthy T cells **(A)**, ARPC1B deficiency **(B)**, HEM1 deficiency **(C)**, WASP deficiency **(D)**, WIP deficiency **(E)**, and WDR1 deficiency **(F)**. Scale bar: 10 μm.

As described above, distinct but interconnected actin networks sustain IS assembly and dynamics (Figure 2, central panel). The molecular machinery that orchestrates this complex combination of local actin networks has started to be elucidated. In particular, research on actin-related PID is pointing to the molecules that play an essential role in this process and helps bridging molecular defects at the IS to downstream impairments in T cell function (see Table 1). To date, T cell synapse defects have been characterized in 5 PIDs caused by defects in actin-binding proteins (ARPC1B, HEM1, WASP, WIP, and WDR1), with the overall finding that the corresponding proteins play non-redundant roles at the IS. As summarized in Figure 2, we review here current knowledge on the molecular activities of these 5 regulators, in terms of actin remodeling and T cell IS microarchitecture.

**TABLE 1:** Inventory of immune-related actinopathies and associated T cell synapse defects.

**Actin-related inborn errors of immunity**				**T cell synapse defects**			
**Gene**	**Protein**	**Initial description**	**Clinical symptoms**	**Patient T cells**	**References**	**Cellular and murine models**	**References**
*ACTB*	β-actin	Nunoi et al., 1999	Mental retardation, recurrent bacterial and viral infections	n.t.		n.t.	
*ARHGEF1*	ARHGEF1	Bouafia et al., 2019	Airways infections, defective antibody production	n.t.		n.t.	
*ARPC1B*	ARPC1B	Kahr et al., 2017; Kuijpers et al., 2017; Somech et al., 2017	Failure to thrive, platelet abnormalities, eczema, infections, vasculitis, hepatosplenomegaly, thrombocytopenia	Deficient cell spreading during IS assembly and formation of spike-like structures	Brigida et al., 2018	CK666 treatment induces collapse of branched actin network and increases linear actin filaments	Murugesan et al., 2016
*CARMIL2*	CARMIL2	Sorte et al., 2016; Wang et al., 2016; Schober et al., 2017	Malignancy (EBV+), IBD, recurrent skin and upper airway infections, failure to thrive	n.t.		No modification of IS formation, but absence of CD28-CARMIL2-CARM1 colocalization	Liang et al., 2013; Roncagalli et al., 2016
*CDC42*	Cdc42	Takenouchi et al., 2015; Gernez et al., 2019; Lam et al., 2019; Szczawinska-Poplonyk et al., 2020	Autoinflammation, HLH, malignant lymphoproliferation	n.t.		Impaired actin remodeling at the IS	Chemin et al., 2012
*CORO1A*	Coronin-1A	Shiow et al., 2008	Bacterial and viral infections, aggressive EBV-associated B cell lymphoproliferation, T cell lymphopenia, T-B+ SCID	n.t.		Increase of WASP and ARP2/3 at the IS area, accumulation of F-actin	Föger et al., 2006; Mugnier et al., 2008
*DIAPH1*	DIAPH1/mDIA1	Kaustio et al., 2021	seizures, cortical blindness, microcephaly syndrome (SCBMS), mitochondrial dysfunction and immunodeficiency	Reduced adhesion and MTOC polarization to the IS, attenuated mitochondrial calcium	Kaustio et al., 2021	Centrosome polarization, spatiotemporal control of Zap70-dependent LAT phosphorylation	Gomez et al., 2007; Thumkeo et al., 2020
*DEF6*	DEF6	Serwas et al., 2019	Severe autoimmune manifestations, recurrent infections	n.t.		Impaired actin polymerization upon TCR activation, defective ZAP-70 polarization at the IS area	Fanzo et al., 2006
*DOCK2*	DOCK2	Dobbs et al., 2015	Severe invasive bacterial and viral infections	n.t.		Defect in IS formation and size, impaired TCR and lipid raft translocation upon TCR engagement	Sanui et al., 2003; Le Floc’h et al., 2013
*DOCK8*	DOCK8	Engelhardt et al., 2009; Zhang et al., 2009, 8	Upper airway infections, susceptibility to viral infection	n.t.		Defective IS formation and reduction of LFA-1 recruitment at the IS area	Randall et al., 2011
*NCKAP1L*	HEM1	Castro et al., 2020; Cook et al., 2020; Salzer et al., 2020	Fever, recurrent bacterial and viral skin infections, severe respiratory tract infections, poor antibody responses, autoimmune manifestations	Dismorphic IS structure, preserved actin foci, LFA-1 activation and conjugate formation towards target cells	Cook et al., 2020; Salzer et al., 2020	Impaired localization of LFA-1 and β1 integrins at the IS	Nolz et al., 2007
*MKL1*	MKL1	Record et al., 2015	Severe bacterial infections, skin abscesses	n.t.		n.t.	
*MSN*	MOESIN	Lagresle-Peyrou et al., 2016	Eczema, episodic bacterial and VZV infections, lymphopenia	No alteration of IS	Lagresle-Peyrou et al., 2016	Treatment with calyculin A induces inhibition of TCR clustering, absence of F-actin exclusion	Ilani et al., 2007
*MYH9*	Myosin IIA	Seri et al., 2003	MHY9-related diseases: May-Hegglin anomaly, Sebastian syndrome, Fechtner syndrome, and Epstein syndrome; mild macrothrombocytopenia, leukocyte inclusions	n.t.		Failure to assemble pSMAC and cSMAC, defective IS stability, modification of TCR microcluster velocity and directionality	Ilani et al., 2009; Kumari et al., 2012; Yi et al., 2012
*NIK*	NIK	Willmann et al., 2014	CVID, recurrent bacterial and viral infections, candidiasis	n.t.		Decreased expression of proteins involved in IS formation and F-actin dynamics	Lacher et al., 2018
*PSTPIP1*	PSTPIP1	Wise et al., 2002	Oligoarticular pyogenic arthritis, acne, pyoderma gangrenosum-like lesions	Defective IS assembly and stability	Janssen et al., 2018	Defect of IS assembly	Badour et al., 2003
*RAC2*	RAC2	Ambruso et al., 2000	Lymphopenia, recurrent respiratory infections, poor wound healing, leukocytosis	n.t.		n.t.	
*RASGRP1*	RASGRP1	Salzer et al., 2016; Winter et al., 2018	Severe pneumonia, failure to thrive, EBV susceptibility	n.t.		n.t.	
*RHOG*	RhoG	Kalinichenko et al., 2021	HLH features, fever, cytopenia, low hemoglobin	Decreased F-actin density at the IS	Kalinichenko et al., 2021	n.t.	
*RHOH*	RhoH	Crequer et al., 2012b	Persistent EV-HPV infections, skin lesions	n.t.		RhoH overexpression increases conjugate formation; defective recruitment of Lck and ZAP70	Chae et al., 2010; Baker et al., 2012
*STK4/MST1*	MST1	Crequer et al., 2012a; Nehme et al., 2012	Recurrent infections, EBV infections, skin lesions and infections	n.t.		Decrease IS formation and stability, severe reduction of T-APC stable contacts, mislocalization of kindlin3 at the IS	Tomiyama et al., 2013; Kondo et al., 2017
*WAS*	WASP	Derry et al., 1994	Thrombocytopenia, eczema, T cell lymphopenia, recurrent bacterial and viral infections	Aberrant actin protrusions, unstable IS, reduced LFA1 clustering	Dupré et al., 2002; Calvez et al., 2011; Houmadi et al., 2018	Unstable IS	Sims et al., 2007
*WDR1*	WDR1	Kuhns et al., 2016; Standing et al., 2017; Pfajfer et al., 2018	Autoinflammation, skin and airway infections	Actin accumulation and disorganized IS structure	Pfajfer et al., 2018	n.t.	
*WIPF1*	WIP	Lanzi et al., 2012	Eczema, T lymphopenia, thrombocytopenia,	Aberrant spreading in the context of the IS	Pfajfer et al., 2017	n.t.	

**n.t., not tested.**

#### WASP Deficiency

The Wiskott-Aldrich syndrome (WAS) is an X-linked PID characterized by microthrombocytopenia, eczema, recurrent infections and increased risk of autoimmune manifestations and malignancies (Ochs and Thrasher, 2006). This severe disorder is caused by a combination of cellular defects in all hematopoietic cell subsets, in accordance with the expression profile of the corresponding protein WASP. However, intrinsic defects of T cell subsets expressing non-functional mutated WASP or lacking WASP, including effector CD4^+^ T cells (Trifari et al., 2006) and CD8^+^ T cells (De Meester et al., 2010), Treg (Adriani et al., 2007; Humblet-Baron et al., 2007; Maillard et al., 2007; Marangoni et al., 2007) and Tfh (Zhang et al., 2016) are sought to contribute to susceptibility to infections, tumors and auto-immune manifestations described to occur at high frequency in WAS patients. In addition, there is a clear selective advantage of WASP^+^ cells over WASP^–^ cells in patients with de novo corrective mutation (Ariga et al., 2001; Wada et al., 2001; Trifari et al., 2010). This observation has contributed to build the rational for the implementation of gene therapy, which is now proposed as an alternative to HSC-T for the treatment of WAS (Aiuti et al., 2013). WASP has been recognized early as an effector of Cdc42 promoting actin polymerization via the Arp2/3 complex (Symons et al., 1996; Machesky and Insall, 1998). WASP has been among the first actin regulators to be identified as a key regulator of IS assembly (Dupré et al., 2002; Orange et al., 2002; Sasahara et al., 2002). Follow-up studies have helped understanding that WASP is predominantly involved in the stabilization of the IS, rather than its initial assembly (Sims et al., 2007). T cells derived from WAS patients appear indeed to assemble kinapses rather than synapses when contacting APC (Calvez et al., 2011). This bias towards kinapses has been attributed to an inability to mediate the stop signal, especially in the context of APC presenting low antigen concentrations (Cannon and Burkhardt, 2004; Lafouresse et al., 2012). Interestingly, the motile synapses of WASP-deficient T cells have been associated to dispersed TCR triggering and erratic calcium flux, which may underline biased defects in Th1 cytokine via delayed NFAT-1 nuclear translocation and defective T-bet induction (Cianferoni et al., 2005; Trifari et al., 2006; Taylor et al., 2010; Calvez et al., 2011). WASP appears to play a direct role in TCR signal organization since it promotes the assembly of dense actin foci associated with TCR microclusters, together with Arp2/3 (Kumari et al., 2015). Such TCR-driven actin foci may provide a platform for TCR signalosome organization and concomitantly a structure to potentiate interaction with the APC via invadopodia-like membrane protrusions. Indeed, in the absence of WASP, TCR-driven actin foci assembly and phosphorylation of the mechanosensory molecule CasL are severely impaired (Kumari et al., 2015). WASP was also shown to mediate via Nck the cross-linking to the actin cytoskeleton of molecular condensates containing the key signaling molecules LAT, Grb2, Sos1, and SLP-76 (Ditlev et al., 2019). Those condensates were shown to dependent on actin coupling for their centripetal diffusion at the dSMAC, while they seemed to uncouple form WASP to cross the pSMAC presumably because of heightened formin activity. Such molecular condensates might be associated to the WASP-dependent actin foci.

Inability of WASP-deficient T cells to maintain stable IS is also relevant to CD8^+^ T cells. WAS CD8^+^ T cells display reduced killing activity while their ability to secrete lytic granules is maintained (De Meester et al., 2010). Live microscopy recording of CTL and target cell interactions revealed a delay in the ability of WASP-deficient CTL to deliver the lethal hit (Houmadi et al., 2018). WASP-deficient CTL were observed by TIRF microscopy to form unstable synapses in association with an actin cytoskeleton meshwork of reduced density. At the nanoscale, WASP-deficiency was associated with reduced number and molecular density of LFA-1 nanoclusters. At the cellular scale, this was associated with a reduced ability to maintaining the LFA-1 adhesive ring (Houmadi et al., 2018), which plays a key role in CD8^+^ T cells to allow polarized delivery of lytic granule content (Somersalo et al., 2004). Interestingly, the role of WASP in controlling IS stability also applies in the context of IS turnover, a poorly studied step of the IS life cycle (Kumari et al., 2020). IS decay has indeed been shown to be directly related to the degradation of WASP as part of ubiquitination and proteolysis mechanisms. This suggests that WASP may act as a molecular timer in the context of T cell stimulation, with its activation leading to actin foci assembly and IS stabilization, but also programming it for degradation with IS destabilization as a consequence.

#### WIP Deficiency

Wiskott-Aldrich syndrome protein (WASP)-interacting protein (WIP) was initially identified with a yeast two-hybrid system aiming at identifying WASP partners (Ramesh et al., 1997). WIP deficiency in humans appears to be extremely rare. The few identified patients with WIP deficiency carry severe mutations leading to undetectable protein expression (Lanzi et al., 2012; Pfajfer et al., 2017). WIP deficient patients present with a WAS-like phenotype, including eczema, T cell lymphopenia and recurrent infections. Because WIP acts as a chaperone for WASP (de la Fuente et al., 2007), WIP deficiency leads to defective expression of WASP, which explains the partial overlap of phenotypes between the 2 deficiencies. CD8^+^ T cells and NK cells from WIP-deficient patients harbor cytotoxic defects that are related to defective contact with target cells. WIP-deficient CD8^+^ T cells display a very aberrant actin organization in the context of IS assembly, which suggests additional defects than those caused by the lack of WASP (Pfajfer et al., 2017). The actin network in WIP deficient T cells is poorly reticulated indicating reduced Arp2/3-dependent actin branching activity. A possible explanation for this severe phenotype is the dual role of WIP in promoting Arp2/3 activation via WASP and cortactin (Kinley et al., 2003). In the context of the IS, WIP is enriched in the peripheral lamellipodia and along the cell body cortex. Structured illumination microscopy revealed that it distributes as tiny patches that intermingled the reticulated actin network of the IS periphery (Pfajfer et al., 2017), in agreement with a role in promoting densely reticulated actin networks via Arp2/3 branching.

#### ARPC1B Deficiency

ARPC1B is part of the Arp2/3 complex, which is the molecular unit that promotes the branching of new actin filaments on the side of mother filaments. The ARPC1 subunit exists under 2 isoforms, ARPC1A and ARPC1B. The ARPC1B subunit is exclusively expressed in the hematopoietic lineage. The discovery of ARPC1B deficiency in humans is fairly recent (Kahr et al., 2017; Kuijpers et al., 2017; Somech et al., 2017). Patients suffer from combined immunodeficiency, allergy and inflammation. In particular patients are subject to severe and prolonged lung viral infections. A number of these patients also display severe bleeding and skin vasculitis (Volpi et al., 2019). Comparably to WASP deficiency ARPC1B deficiency impacts thrombocyte development, leading to microthrombocytes, as well as neutrophil motility (Volpi et al., 2019). ARPC1B also plays a crucial role in the T cell compartment since patients harbor defects in T cell proliferation, migration, and cytotoxic activity and T_*REG*_ functions (Brigida et al., 2018). Upon IS assembly in healthy T cells, the Arp2/3 complex polarizes to the peripheral lamellipodia (Brigida et al., 2018). In ARPC1B deficient patients, T cells fail to emit a lamellipodia structure. They instead emit aberrant actin-rich structures, including spikes and long filopodia-like structures, both in the context of 2D IS and contact with APC (Brigida et al., 2018; Randzavola et al., 2019). As a result of such defective IS, T cells from ARPC1B deficient patients form less stable conjugates and kill less efficiently. Additionally to its role in driving IS assembly via the lamellipodium, ARPC1B has been proposed to control the recycling of key molecules such as the TCR, CD8 and GLUT1, via the retromer and the WASH complex (Randzavola et al., 2019). Accordingly the CD8^+^ T cells from an ARPC1B deficient patient were found to express low levels TCR, CD8 and GLUT1, implying that the functional defects of these cells arise from a complex combination of defects of multiple actin networks. This is in line with the pleiotropic activity of the Arp2/3 complex in multiple specialized actin networks.

#### HEM1 Deficiency

Hematopoietic Protein 1 (HEM1), which is encoded by the ***NCKAP1L*** gene, is part of the pentameric WAVE complex, which represents an alternative activator of the Arp2/3 complex in parallel to WASP. Comparably to WASP, HEM1 is an hematopoietic specific protein reinforcing the notion that immune cells are equipped with distinct activators of the Arp2/3 complex. The WAVE complex exists in an autoinhibitory state in the cytoplasm. Its activation depends on the coincident activity of prenylated Rac1 and association to membranes containing acidic phospholipids (Lebensohn and Kirschner, 2009). In particular the 3D opening of WAVE2 frees its VCA domain, which promotes interaction with the Arp2/3 complex and drives actin branching. HEM1 deficiency in humans has been described recently in 3 independent studies (Castro et al., 2020; Cook et al., 2020; Salzer et al., 2020). Patient-derived T cells display reduced F-actin density associated with a dysmorphic IS structure. These cells fail to assemble the typical actin ring and instead display an excess of spikes proposed to be formin-dependent. However, HEM1-deficient T cells still possess actin foci, suggesting that WASP activity is maintained in these cells. The WAVE complex appears to locate primarily at the periphery of the lamellipodia, implying that its main function is to promote lamellipodia growth via Arp2/3. Previous studies in mice with WAVE2 deficiency have pointed to the role of the WAVE complex in CRAC-mediated calcium entry (Nolz et al., 2006) and integrin activation (Nolz et al., 2007). Whether this applies to HEM1 deficient human T cells remains to be explored. Although Hem1-deficient CD8^+^ T cells display severe IS defects, this does not appear to translate in cytotoxic T cell dysfunction, at least as measured *in vitro*. Interestingly, these cells appear to secrete abnormally high amount of lytic molecules following non-specific activation via IL-2 or PMA/ionomycin, probably as a result of the poorly structured cortical actin network whose filtering function might be altered (Cook et al., 2020). However, in the context of specific activation via the TCR, lytic granules secretion in HEM1-deficient cells is similar to that of control cells. It is therefore possible that in the context of HEM1-deficient CD8^+^ T cells, cytotoxic activity might be maintained because facilitated secretion compensates for the aberrantly organized IS. Following TCR stimulation HEM1-deficient T cells harbor defects in upregulation of activation markers and reduction of proliferation and cytokine production (Cook et al., 2020). However such defects might not be solely explained by defective IS. Indeed, in addition to actin remodeling defects, the absence of the HEM1 protein causes a defective activation of the mTORC2/AKT pathway, with HEM1 activating mTORC2 enzymatic activity independently from its association to the WAVE complex. The relative contribution of actin-dependent and actin independent functions of HEM1 to the T cell defects remains to be elucidated.

#### WDR1 Deficiency

WD Repeat Domain 1 (WDR1, also referred as AIP1 for Actin-Interacting Protein 1) acts in concert with cofilin and coronin to promote actin filament disassembly via a severing activity. This process is essential for fast actin filament turnover (Kotila et al., 2019). WDR1 deficiency is a recently described PID which leads to inflammation, multiple respiratory tract infections and impact both innate and adaptive immunity (Kuhns et al., 2016; Standing et al., 2017; Pfajfer et al., 2018). Regarding the lymphoid compartment, the B cells are more affected by the WDR1 mutants than T cell compartment, depicted by a paucity of B cell in the bone marrow and in the periphery. While T cell development seems to be normal in patients, WDR1 deficiency results in a reduction of the T_*FH*_ subset, most likely due to B cell lymphopenia (Pfajfer et al., 2018). T cells, but also neutrophils, monocytes and B cells, present an accumulation of F-actin content, more probably due to a defect in actin severing/depolymerization, as it has been described in mice (Kile et al., 2007). In the context of the IS, WDR1-deficient T cells harbor a variety of actin-rich structures usually not encountered in healthy primary T cells, such as actin arcs, actin spikes and large actin condensates (Pfajfer et al., 2018). Some of those actin structures and in particular arcs are reminiscent of the formin-dependent structures described to predominantly occur in Jurkat T cells (Murugesan et al., 2016). This suggests that reduced actin turnover in WDR1-deficient T cells might shift the equilibrium between the Arp2/3-driven branching and formin-driven elongation activities, in favor of the latter. Unexpectedly however, only minor alterations of T cell activation were detected in the primary T cells isolated from the studied WDR1-deficient patients. A mild reduction of the calcium response upon TCR activation was measured, while proliferation, motility, cytotoxicity and TCR internalization were preserved (Pfajfer et al., 2018). Of note WDR1 deficiency appeared to more severely affect the B cell compartment. Why WDR1 deficiency differentially affects the activation and differentiation of T cells and B cells is currently unknown.

We have reviewed above how natural deficiencies are contributing to revealing the role of key individual actin-binging proteins in T cell IS assembly. From these studies, it emerges that the corresponding proteins play non-redundant function in IS assembly, with their deficiencies leading to characteristic defects. It is particularly striking that HEM1 and WIP are required for IS spreading via lamellipodia extension, while WASP seems to be particularly involved in IS stability and turn-over. WDR1 deficiency highlights distinct requirements of T and B cell IS of actin turn-over for IS function. Can the comparative analysis of these actinopathies tell us about how IS controls T cell activation and function? Interestingly CD8^+^ T cells from these patients display distinct IS defects but also a wide range of cytotoxic impairment (from non-affected in HEM1 deficiency, to mildly reduced in WAS to more severely affected in WIP and ARPC1B deficiencies). The precise nature of the actin cytoskeleton alterations rather than the apparent severity of IS morphology seems to account for functional outcome. In addition, complex combinations of defects (such as discussed for HEM1 deficiency) renders prediction of how IS impairments may translate in defective function difficult to make.

### Immunological Synapse Defects in Additional Actin Related Deficiencies

**FIGURE 3:**
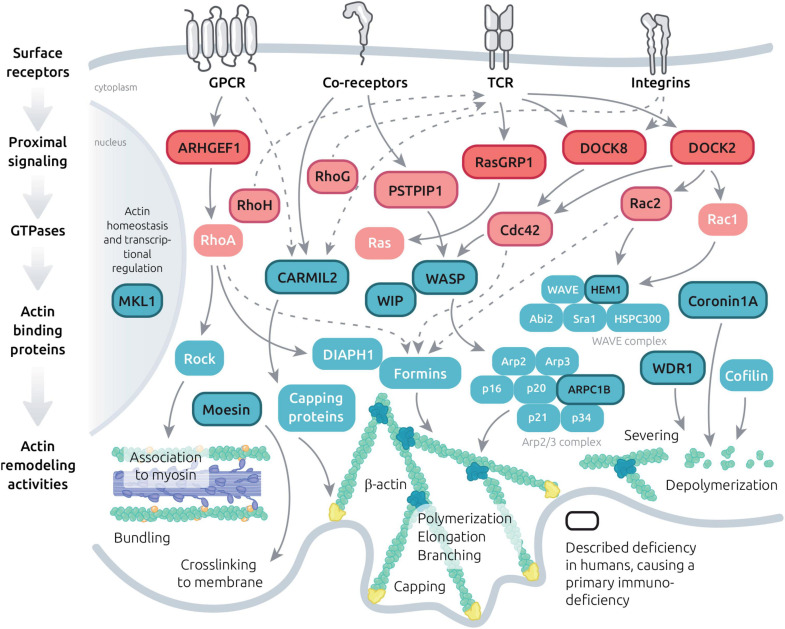
Molecular machinery controlling actin dynamics in T cells. Schematic view of the major signaling events leading to actin cytoskeleton remodeling downstream key T cell surface receptors. The represented molecules include GTPase regulators, GTPases and actin binding proteins with diversified actin remodeling activities. A focus is made on molecules which have been implicated in PID (dark letters and lines) and on their interactors.

Beyond the 5 actin-related PIDs described in detail above, additional 15 monogenic immunodeficiencies related to the actin remodeling machinery have been characterized to date (Figure 3 and Table 1). This includes deficiency in the actin-binding proteins coronin-1A (Shiow et al., 2008), moesin (Lagresle-Peyrou et al., 2016) and CARMIL2 (Sorte et al., 2016; Wang et al., 2016; Schober et al., 2017). In addition, a point mutation in the β-actin gene (*ACTB*) itself has been reported in a single patient presenting with immunodeficiency, thrombocytopenia, but also intellectual and developmental impairments (Nunoi et al., 1999). More upstream in the actin remodeling machinery, deficiencies in the Rho GTPases Rac2 (Ambruso et al., 2000), RhoH (Crequer et al., 2012b), Cdc42 (Takenouchi et al., 2015; Gernez et al., 2019; Lam et al., 2019; Szczawinska-Poplonyk et al., 2020) and RhoG (Kalinichenko et al., 2021) have been characterized in patients with various forms of immunodeficiency. Actin-related PIDs also include deficiencies in the GTPase regulators DOCK2 (Dobbs et al., 2015), DOCK8 (Engelhardt et al., 2009; Zhang et al., 2009), DEF6 (Serwas et al., 2019) and ARHGEF1 (Bouafia et al., 2019). Finally, deficiencies in more indirect regulators of actin cytoskeleton dynamics have been associated to PID entities. This includes CD2BP1/PSTPIP1 (Wise et al., 2002), MST1/STK4 (Crequer et al., 2012a; Nehme et al., 2012), NIK (Willmann et al., 2014), MKL1 (Record et al., 2015) and RasGRP1 (Salzer et al., 2016; Winter et al., 2018). These PID-related proteins build a molecular network that covers key aspects of the actin remodeling machinery (Figure 3). Given the fundamental role of the actin cytoskeleton in sustaining multiple processes occurring at the IS, it is conceivable that most of the above-listed PID-related regulators concur to drive actin dynamics in the context of the T cell IS. Indeed, complementary studies in murine models or model cell lines are pointing to the contribution of multiple additional molecular actors, as depicted in Figure 3. For some of the actin-related PIDs, it should be noted that IS defects in NK cells and B cells have also been characterized, although these are not covered in the present review. We focus below on the 3 actin-binding proteins coronin-1A, moesin and CARMIL2.

#### Coronin-1A Deficiency

Coronin-1A is an hematopoietic cell-specific member of the conserved coronin family, which binds F-actin via WD repeat domains. Coronin-1A interacts with the Arp2/3 complex and inhibits its activity (Humphries et al., 2002). It also promotes actin disassembly in association with cofilin and WDR1. Mutations in *CORO1A*, the gene that encodes coronin-1A, lead to a severe form of PID with life-threatening infections and EBV-driven lymphoproliferation (Shiow et al., 2008; Moshous et al., 2013). This deficiently particularly affects the T cell compartment with severe T cell lymphopenia related to impaired egress from the thymus, in line with the role of coronin-1A in T cell trafficking (Föger et al., 2006). Beyond this defect, peripheral T cells have been shown to depend on coronin-1A for Ca^2+^ mobilization from intracellular stores (Mueller et al., 2008). This activity depends on coronin-1A interaction with PLC-γ1 and might be independent from its actin remodeling one. Furthermore coronin-1A has been shown to regulate cAMP levels in peripheral T cells (Jayachandran et al., 2019), reinforcing the notion that this protein exerts actin-independent functions in T cells. How the actin-dependent function of coronin-1A might regulate T cell IS assembly and dynamics has started to be investigated in CD4^+^ murine cells (Britton et al., 2017). In those cells, PKCθ keeps coronin-1A in a phosphorylated inhibitory state at the IS center. Where at the T cell IS and when coronin-1A may exerts its Arp2/3 inhibitory and actin disassembly activities still remain open questions. The actin-related role of coronin-1A at the IS has been clarified in more detail in the context of NK cells. In particular the study of a coronin-1A-deficient patient has highlighted that coronin-1A is indispensable for NK cell cytotoxic activity (Mace and Orange, 2014). Such defect was found to be related to a defective deconstruction of the cortical actin meshwork, which is required for lytic granule secretion. Whether a similar process is operating in cytotoxic T cells has not been explored yet.

#### Moesin Deficiency

As introduced above, the ERM proteins crosslink the actin cytoskeleton to the plasma membrane So far, only moesin among the ERM family has been reported to be associated with primary immunodeficiencies. Human moesin deficiency affects particularly the T cell compartment, resulting in T cell proliferation and migratory defects (Lagresle-Peyrou et al., 2016; Delmonte et al., 2017). Moesin-deficient human T cells fail to exclude CD43 molecules (Lagresle-Peyrou et al., 2016). Indeed, upon TCR activation, moesin is dephosphorylated, and loses its ability to binds CD43 molecules, which are not anchored to the actin cytoskeleton anymore (Delon et al., 2001; Ilani et al., 2007). CD43 molecules are then supposedly more motile and are excluded from the IS area. After roughly 20 min after TCR activation, moesin molecules have been rephosphorylated, resulting in a rapid reattachment of CD43 molecules at the pSMAC, leading to the stable localization of CD43 molecules at this site (Delon et al., 2001; Brown et al., 2003). It remains to be investigated which other molecules might be affected in their dynamics relocation in the context of moesin deficiency. How the anchoring function of moesin affects downstream signaling events and globally controls T cell activation and function remains to be explored.

#### CARMIL2 Deficiency

CARMIL2 (also known as RLTPT in mice) is a capping protein expressed in the lymphoid compartment. Patients carrying CARMIL2 mutations present recurrent upper airway infection and are more susceptible to EBV infection leading to disseminated EBV^+^ smooth muscle tumors (SMT) (Schober et al., 2017). CARMIL2 deficiency leads to a combined immunodeficiency, in which the T cell compartment is particularly affected. This is explained by the critical role of CARMIL2 in mediating CD28-evoked costimulation. In mirror with mice study (Liang et al., 2013), CARMIL2 deficiency leads to defective T_*REG*_ development as well as defective CD28-mediated T cell function (cytokine production, proliferation). In addition, T cells present a defective migration capacity and directionality due an abnormal actin structure at the leading edge (Schober et al., 2017). So far, the role of CARMIL2 protein in IS assembly has not been explored. A mice study described a defect of PKCθ clustering in T cells where Rltpr is mutated to block the CD28 signaling pathway (Liang et al., 2013). This shows that in normal T cells, PKCθ is recruited at the IS following TCR engagement, and is dependent of the CD28-CD80 binding and Rltpr. However, reported CARMIL2-deficient patients did not show defective PKCθ phosphorylation upon TCR stimulation.

### Formins as Important Regulators of the T Cell Synapse

Beyond the molecules studied in the context of actin-related PIDs, additional actin regulators have been shown to be involved in IS assembly and regulation. A particularly pivotal role in actin cytoskeleton remodeling is played by the formin family comprises 15 members in mammals that promote linear actin filament polymerization by recruiting profilin-bound actin monomers and protecting elongated actin filaments from proteins with anti-capping activity (Breitsprecher and Goode, 2013). Formins play essential roles in cell migration, adhesion, and cell-cell interactions. As such they are particularly relevant to sustaining immune cell function. T cells express at least 2 members of the formin family, Diaphanous-related formin-1 (mDIA1, DIAPH1), and Formin-like 1 (FMNL1, FRL1) (Gomez et al., 2007). Interestingly, mDIA1 and FMNL1 carry GTPase-binding domains for RhoA and Cdc42, respectively (Schönichen and Geyer, 2010), suggesting distinct connectivity to acto-myosin and actin remodeling activities. Studies in mDIA1 KO mice have revealed the role of mDIA1 in promoting T cell emigration from the thymus and recruitment into peripheral tissues (Vicente-Manzanares et al., 2003; Eisenmann et al., 2007; Sakata et al., 2007). More recently, FMNL1 has been shown to mediate effector T cell trafficking to inflammatory sites in the context of T cell-mediated autoimmunity, by promoting actin polymerization at the back of the nucleus to help T cells overcome restrictive barriers (Thompson et al., 2020). In the context of the T cell IS both mDIA1 and FMNL1 contribute to the reorientation of the MTOC and their deletion in CD8^+^ T cells reduces cytotoxic activity (Gomez et al., 2007). These 2 formins appear to have distinct localization in the lamellipodium of T cells forming the IS. Moreover, FMNL1 and mDIA1 are enriched around the MTOC, arranging into tight ring and starburst patterns, respectively (Gomez et al., 2007). This suggests that mDIA1 and FMNL1 play complementary roles in driving MTOC polarization in T cells upon cognate APC encounter. Whether these roles are exerted directly or via the peri-centriole actin network described recently at the B cell synapse (Obino et al., 2016) remains to be investigated. A further layer of complexity regarding formin activities in the context of T cell activation is the existence of 3 FMNL1 isoforms (FMNL1α/β/γ) with distinct C-term domains that appear to distribute differentially at the MTOC, actin cortex and golgi apparatus (Colón-Franco et al., 2011; Bello-Gamboa et al., 2020). Recently, mDIA1 and mDIA3 were found to play a key role at the T cell IS by controlling a very early step of TCR signaling. Indeed ablation of these formins in murine CD8^+^ T cells affected the localization of phosphorylated Zap70 and downstream LAT phosphorylation (Thumkeo et al., 2020). These formins were shown to also be indispensable for IS spreading, peripheral F-actin ring formation and TCR microcluster centralization. These findings point to the very proximal roles for F-actin remodeling in the context of TCR-driven antigen recognition, as discussed in chapter 1.

There is to date no reported PID entity that would be caused by genetic alterations in genes encoding formins. However, homozygous loss of *DIAPH1* (the gene that encodes DIAPH1/mDIA1), which was initially reported to cause is causing seizures, cortical blindness, and microcephaly syndrome in humans (Ercan-Sencicek et al., 2015), has recently been reported to be associated with lymphocyte maturation and function impairments, as well as increased risk of B cell lymphoma (Kaustio et al., 2021). The immune cell defects included defective T cell IS assembly, in particular MTOC polarization, in agreement with earlier cell biology studies. The severity of the neurological manifestations related to the role of mDia1 in brain development have initially masked immune system deficiency in the affected patients. Furthermore, a gain-of-function variant in *DIAPH1* was reported to cause hearing loss associated to macrothrombocytopenia and mild neutropenia (Stritt et al., 2016). Lymphocyte function was reported to be abnormal in these patients. It is possible that specific mutations in additional formin isoforms with preponderant expression in the hematopoietic system will be identified as causative in yet undiagnosed PIDs or complex syndromes.

## Actin Remodeling Program Sustaining Immunological Synapse Assembly and Turnover

### The Actin Remodeling Machinery in the Context of T Cell Activation

The knowledge gained through the study of individual actinopathies and complementary models is providing the notion that actin regulators play non-redundant activities in the context of T cell activation and function (as illustrated in Figure 2). Beyond the study of single deficiencies, a yet poorly studied question is that of the coordination at work among such regulators. Indeed, actin cytoskeleton homeostasis relies on a coherent molecular program to guarantee coexistence of multiple actin networks, their adaptability to stimuli and their turnover. Actin regulators are integral to the signaling events provoked upon engagement of the TCR, integrins, coreceptors, chemokine receptors (Figure 3) and probably nearly any T cell surface receptor. In a global atlas of TCR signaling, the earliest signaling events have recently been mapped to proteins belonging to the term “guanine nucleotide releasing factors,” “GTPase activation,” “cell projection,” “cytoskeleton,” and “actin binding” (Locard-Paulet et al., 2020). This makes sense given that actin remodeling is required for synapse initiation and spreading, as well as to drive receptor patterning. As illustrated in Figure 3, the layers connecting surface receptors to the various actin remodeling activities encompass proximal signaling molecules, GTPase regulators, GTPases and actin binding proteins. The hierarchical orchestration of this signaling network in time and space is barely understood. Beside basic studies conducted in various cellular models, studies on the immune-related actinopathies presented above have started to provide original insight into specific facets of the molecular interplay sculpting the actin remodeling ensemble (in Figure 3, molecules related to PID have been highlighted). We will focus in the next chapter on the molecular details about the WIP-WASP-DOCK8 interaction, since more knowledge has been accumulated for this area of the actin remodeling network (at least in the context of T cells). It provides a glimpse into the nature of the connectivity between TCR stimulation and actin remodeling.

### Complex Coordination Among Actin Regulators: Focus on WIP-WASP-DOCK8

As explained above, WIP has initially been described as a chaperone for WASP. Accordingly, WIP-deficient T cells have been reported to fail expressing WASP, both in mice models and corresponding patients (de la Fuente et al., 2007; Lanzi et al., 2012; Pfajfer et al., 2017). The loss of WASP expression in the context of WIP deficiency can be explained by the fact that WIP protects WASP from ubiquitination and its degradation by the proteasome and calpain (Chou et al., 2006; de la Fuente et al., 2007). In addition to protecting WASP from degradation, WIP has been shown in non-hematopoietic cells to act as a link between Nck and N-WASP (Donnelly et al., 2013), the homologue of WASP in non-hematopoietic cells. This positions WIP as a molecule capable to connect signaling events proximal to receptors to WASP/N-WASP-dependent actin remodeling. The hierarchy of connections between TCR stimulation and actin remodeling and the pivotal role of WIP has been recently revealed by a phosphoproteomic approach showing that WIP phosphorylation events are among the earliest marks upon TCR stimulation (Locard-Paulet et al., 2020). Indeed, WIP belongs to a cluster of signaling molecules with very rapid but transient phosphorylation, with Serine 268 and 330 phosphorylated as early as 15 sec after TCR stimulation. Interestingly Serine 340 of WIP is phosphorylated at a later time point and in a prolonged manner. This suggests a chronological orchestration of signaling events along the steps of IS maturation. Those molecular marks are not within the WASP interaction domain and may not directly impact WIP activity towards WASP. Rather, PKCθ phosphorylation of WIP at S488 has been proposed as a mediator of is accompanied by the dissociation of the WIP/WASP complex dissociation, which is a pre-requisite required for WASP activation (Sasahara et al., 2002; Fried et al., 2014b).

A further layer of complexity of the WIP-WASP pathway relates to the WASP-independent functions of WIP. T cell synapse defects appear to be more severe in WIP-deficiency as they are in WASP-deficiency (Calvez et al., 2011; Pfajfer et al., 2017), although these 2 deficiencies have not formally been compared side by side with similar models and stimulations. The distinct severity of IS defects (as well as other defects in T cells and other cell types, as reviewed in Fried et al., 2014a) can be explained by the fact that the function of WIP is not limited to protecting WASP from degradation. Because of its structure and abundance of proline residues, WIP indeed interacts with multiple proteins including additional actin-related proteins, beyond WASP. This includes cortactin that promotes Arp/2/3-dependent actin polymerization (Kinley et al., 2003). Furthermore, T cells from mice that express a WIP mutant that lacks the capacity to bind actin without affecting WASP levels display decreased cellular F-actin content, a disorganized actin cytoskeleton, impaired chemotaxis and *in vivo* homing (Massaad et al., 2014). Together, these data suggest that WIP controls lymphocyte motility independently from its WASP stabilization function, possibly via its capacity to bind actin directly and to interact with Nck and cortactin (Antón and Jones, 2006).

The interaction of WASP and WIP occurs as part of a molecular complex also including the upstream regulator DOCK8, as shown in T cells by co-immunoprecipitation assays (Janssen et al., 2016). Interestingly WIP bridges DOCK8 to WASP and to actin via distinct molecular domains. Therefore WIP acts as a scaffold with actin affinity, on which WASP and DOCK8 can dock. Such molecular complex assembly allows WASP activation by the GEF activity of DOCK8 (via Cdc42), at the proximity to actin structures in a way to control local remodeling. The DOCK8 mutant (S1827P in DHR2 domain) with loss of GEF activity for Cdc42 but preserved binding to WIP and WASP was used to show that the control of WASP activity is via its GEF activity (Janssen et al., 2016). In the context of LFA-1 activation, the WIP-WASP-DOCK8 complex can associate with talin to bind to the β chain of LFA-1, which promotes actin anchorage and conformational activation (Ham et al., 2013).

Deficiencies in WASP, WIP and DOCK8 share multiple impairments in T cell function, albeit to a different degree. This indicates that although these proteins belong to a common signaling pathway downstream TCR activation, they are additionally endowed with independent activities. Indeed, DOCK8 exerts WASP-independent functions, such as through its association with STAT3, which explains occurrence of Hyper-IgE in DOCK8-deficient individuals (Zhang et al., 2009). Along the same line, WIP exerts WASP-independent functions, such as through cortactin, which may explain higher severity of WIP deficiency as compared to that of WASP.

### Actin Homeostasis Is Driven by Competition Among Actin Networks

The coexistence of dynamic actin networks within the cell has been proposed to be tuned by a global treadmilling process, in which a steady-state amount of polymerizable actin monomers is established by the dynamics of each network (Carlier and Shekhar, 2017). In that context, specific triggers might result in differential partitioning of actin to distinct networks via the relative activation of Rho GTPases. This view is supported by the notion of competition as a basic principle to determine the size of distinct actin networks (Burke et al., 2014; Lomakin et al., 2015; Antkowiak et al., 2019). In particular, this seems to apply to the cortical actin, which has been shown to be regulated by the activities of the Arp2/3 complex and formins, as competitive actin nucleators (Bovellan et al., 2014).

Such concept of competition at the basis of actin homeostasis is important to fully apprehend some of the cellular defects in patients with actinopathies. For example, we found that aberrant spikes and elongated protrusions dynamically assembled at the periphery of ARPC1B-deficient T cells in the context of TCR/LFA-1 activation result from an imbalanced actin dynamics in favor of formins (Brigida et al., 2018). Indeed patient-derived T cells treated with the pan-formin inhibitor SMIFH2 displayed a collapse of the aberrant protrusions. It should be noted, however, that such treatment did not restore normal assembly of the synapse, pointing to the required intrinsic activity of Arp2/3 to drive IS assembly by favoring lamellipodia extension. This observation is in line with a previous work showing that Arp2/3 inhibition in Jurkat cells results in the emission of formin-dependant actin spikes (Murugesan et al., 2016). This data also illustrates that the consequence of a given molecular deficiency might be the combination of the loss of the intrinsic function of the considered actor together with indirect compensatory effects. Another level of co-regulation is illustrated by compensatory mechanisms that might arise in the context of actin-regulator deficiencies. Indeed, ARPC1B deficiency has recently been shown to be accompanied by a molecular compensation via ARPC1A (Randzavola et al., 2019). Why ARPC1A expression does not rescue the function of ARPC1B deficient immune cells remains to be investigated.

### Integration of Concurrent Stimuli by the Actin Remodeling Machinery

Individual studies on actin-related PIDs have been able to pinpoint defects in available cellular models and have focused on a few stimuli. We currently lack information on the selectivity (or ubiquity) of actin remodelers in mediating the activity of the multiple receptors that decorate T cells and contribute to the regulation of migration, adhesion and activation. If one considers WASP, for which more substantial knowledge has been accumulated, studies with patient-derived T cells collectively indicate that it is involved in transmitting activation via multiple receptors including the TCR (Molina et al., 1993; Dupré et al., 2002), CD28 (Badour et al., 2007), integrins (Lafouresse et al., 2012; Houmadi et al., 2018) and chemokine receptors (Haddad et al., 2001). However, the involvement of WASP downstream of many costimulatory or inhibitory receptors has not been explored. Beyond WASP, whether actin regulators tend to share their activity downstream multiple receptors or to operate selectively is currently unknown. Anyway, we favor the view that each T cell triggering (with nature of receptors engaged and strength of engagement as variables) will lead to an adapted remodeling of the local actin networks sustaining the IS. Given the segregation of receptors in distinct areas of the IS that are sustained by distinct cortical actin reticulation, it is tempting to speculate that the different receptors establish distinct crosstalk with the actin cytoskeleton and its remodeling machinery. This notion is supported by the evidence that receptors are organized into distinct membrane subdomains that establish specific connections to the actin cytoskeleton. Indeed, TCR clusters are associated with dense actin foci, which are driven by WASP and Arp2/3 activities (Kumari et al., 2015). In a complementary study, LFA-1 was shown to associate to an actin network regulated by the formin FHOD1 (Tabdanov et al., 2015). Very interestingly, LFA-1 adhesion was shown to mediate actomyosin forces via its associated network, which were necessary to reinforce TCR-associated actin foci. This shows not only that receptors are embedded in specific local actin environments that they shape upon triggering, but also that receptor interplay operates at least in part via connections of these local actin networks. The spatial range at which such crosstalk might operate is currently unknown and will require dedicated micropatterning and imaging approaches, coupled to biosensors able to capture local molecular activities.

## Concluding Remarks

Approaching the molecular control of IS actin dynamics and sub-networks via the prism of actin-related PID has taught us a lot in recent years. Indeed such natural defects have pointed to key molecules, which deficiency translates into pathological states. Beyond the initial studies on actin dynamics that have made use of drugs with crude effects on actin dynamics, understanding of fine molecular control of actin remodeling through precise actin regulators has greatly deepen our understanding of actin homeostasis. With the deployment of genome sequencing, multiple new entities have been discovered recently and will continue to be discovered. Not only does PID research provide molecular pathways of interest, but it also fosters fundamental research in the sense that elucidation of complex deficiencies often challenges the limits of current knowledge.

The morphological analysis of the IS of T cells from patients with deficiencies in WASP, WIP, WDR1, RasGRP1, ARPC1B, and HEM1 reveal distinct roles of individual actin regulators in shaping IS microarchitecture. Together, these data point to the non-overlapping activities of multiple actin regulators during T cell activation, calling for a comprehensive and systematic analysis of T cell signaling in these unique models. Such goal would require better standardization of the current approaches, in particular regarding morphological analysis of the IS (Mace and Orange, 2015; Jankowska et al., 2018). Improving approaches to characterize IS defects might in turn benefit diagnostic efforts and accelerate discovery of yet undiagnosed PIDs related with actin remodeling.

## Author Contributions

LD and LP wrote the manuscript and conceptualized the figures. KB provided intellectual input and edited the manuscript. All authors contributed to the article and approved the submitted version.

## Conflict of Interest

The authors declare that the research was conducted in the absence of any commercial or financial relationships that could be construed as a potential conflict of interest.
